# A comparison between the role of enniatins and deoxynivalenol in *Fusarium* virulence on different tissues of common wheat

**DOI:** 10.1186/s12870-024-04945-5

**Published:** 2024-05-27

**Authors:** Giovanni Beccari, Francesco Tini, Nora A. Foroud, Luisa Ederli, Donald M. Gardiner, Aurelie H. Benfield, Linda J. Harris, Michael Sulyok, Roberto Romani, Ilaria Bellezza, Lorenzo Covarelli

**Affiliations:** 1 Department of Agricultural, Food and Environmental Sciences, University of Perugia, Perugia, Italy; 2 Lethbridge Research and Development Centre, Agriculture and Agri-Food Canada, Lethbridge, Canada; 3 The University of Queensland, St. Lucia, Brisbane, Australia; 4 School of Biomedical Sciences, Faculty of Health, Queensland University of Technology, Translational Research Institute, Brisbane, Australia; 5 Ottawa Research and Development Centre, Agriculture and Agri-Food Canada, Ottawa, Canada; 6 Department of Agrobiotechnology (IFA-Tulln), Institute of Bioanalytics and Agro-Metabolomics, University of Natural Resources and Life Sciences, Vienna, Tulln, Austria; 7 Department of Medicine and Surgery, University of Perugia, Perugia, Italy

**Keywords:** *Fusarium*, Cereals, Mycotoxins, Secondary metabolites, Fusarium head blight

## Abstract

**Background:**

*Fusarium graminearum* and *Fusarium avenaceum* are two of the most important causal agents of Fusarium head blight (FHB) of wheat. They can produce mycotoxins that accumulate in infected wheat heads, including deoxynivalenol (DON) and enniatins (ENNs), produced by *F. graminearum* and *F. avenaceum*, respectively. While the role of DON as a virulence factor in *F. graminearum* toward wheat is well known, ENNs in *F. avenaceum* has been poorly explored. Results obtained to-date indicate that ENNs may confer an advantage to *F. avenaceum* only on particular hosts.

**Results:**

In this study, with the use of ENN-producing and ENN non-producing *F. avenaceum* strains, the role of ENNs on *F. avenaceum* virulence was investigated on the root, stem base and head of common wheat, and compared with the role of DON, using DON-producing and DON non-producing *F. graminearum* strains. The DON-producing *F. graminearum* strain showed a significantly higher ability to cause symptoms and colonise each of the tested tissues than the non-producing strain. On the other hand, the ability to produce ENNs increased initial symptoms of the disease and fungal biomass accumulation, measured by qPCR, only in wheat heads, and not in roots or stem bases. LC-MS/MS analysis was used to confirm the presence of ENNs and DON in the different strains, and results, both in vitro and in wheat heads, were consistent with the genetics of each strain.

**Conclusion:**

While the key role of DON on *F. graminearum* virulence towards three different wheat tissues was noticeable, ENNs seemed to have a role only in influencing *F. avenaceum* virulence on common wheat heads probably due to an initial delay in the appearance of symptoms.

**Supplementary Information:**

The online version contains supplementary material available at 10.1186/s12870-024-04945-5.

## Background

Wheat (*Triticum* spp.) is one of the most cultivated cereals in the world, with a production of about 910 million tonnes in 2021 [1]. Wheat production can be strongly compromised by fungal diseases, such as Fusarium head blight (FHB). FHB is one of the most damaging diseases affecting wheat crops around the world [2]. In addition to yield loss, FHB can adversely affect grain quality due to the accumulation of mycotoxins [3, 4].

FHB is considered a disease complex because it is caused by different species belonging to multiple *Fusarium* species complexes [5]. The composition of the FHB community is dynamic [6] and could be shaped by agricultural practices, susceptibility of cultivated varieties, and climatic conditions (especially during anthesis) as well as fungicide application [7–10]. Globally, *Fusarium graminearum* is considered the most important FHB causal agent due to its high incidence and virulence [11]. *Fusarium culmorum*, *F. avenaceum*, and *F. poae* are also commonly isolated from infected grains and can be found in different regions around the world [12–18]. The incidence of *F. avenaceum* has increased in recent years and is rapidly becoming one of the most common species associated with the FHB complex of wheat and barley in many cultivation areas [14, 19–24]. In addition, this species has also shown frequent co-occurrence with *F. graminearum* [14, 22, 25, 26].

*Fusarium* species associated with FHB are capable of producing a wide range of secondary metabolites that are harmful towards humans and animals [27] and can contaminate raw materials, as well as processed food and feed. Deoxynivalenol (DON), mainly biosynthesized by *F. graminearum* and *F. culmorum*, is the most common *Fusarium* mycotoxin detected in cereals worldwide [28–30].

Due to its occurrence and harmfulness, DON is the most studied and known *Fusarium* mycotoxin [31]. The action of DON toward plants, as well as its role in fungal virulence, has been demonstrated [32]. DON belongs to the trichothecene class of mycotoxins and its production by *F. graminearum* depends on the expression of trichodiene synthase, an enzyme encoded by the *TRI5* gene. This enzyme catalyses the first step in trichothecene biosynthesis, the cyclization of farnesyl pyrophosphate into trichodiene [33]. It is well known that trichothecene toxicity occurs through inhibition of eukaryotic protein synthesis [34] and DON-related damage can be observed at many levels in plants, from cell metabolism, such as inhibition of cell division and mitosis [35, 36], to growth reduction of coleoptiles, roots, and shoot [37–40]. Exogenous applications of DON lead to hydrogen peroxide production, programmed cell death (PCD) as well as a defence response in wheat leaves [41]. Low concentrations of DON can inhibit PCD-disrupting biotrophic-type plant defences [42]. DON is considered a fungal virulence factor as its production is crucial for the spreading of FHB symptoms within wheat heads [43–46]. It is generally thought that the production of DON by *F. graminearum* increases during the switch from its short biotrophic to the necrotrophic phase [47–49], detectable by 24 h post-inoculation and with a significant growth increase by 96 h [50, 51]. This mycotoxin can be transported through vascular elements to the adjacent healthy spikelets, favouring disease development and the typical premature head bleaching, and sometimes associated with the appearance of water-soaked brown, dark purple to black coloured necrotic lesions on the exterior surface of the florets [47, 52, 53].

As an inhibitor of protein synthesis, DON is also dangerous for mammals. It is well-known that its ingestion can result in acute or chronic toxicity, and DON accumulation makes grains inappropriate for human and animal consumption [4, 54]. For this reason, the European Commission (EC) has set the maximum acceptable levels for DON in raw cereals and derived products for human foodstuffs [55].

In recent years, many surveys have shown an increase in the incidence of “emerging mycotoxins” which include several compounds produced by other *Fusarium* species [56]. Enniatins (ENNs) are the most common emerging mycotoxins detected in wheat, barley, and other cereals as well as in cereal products for human and animal consumption [57, 58]. ENNs are mainly produced by members of the *Fusarium tricinctum* species complex (FTSC), which includes *F. avenaceum*, and some members of the *Fusarium sambucinum* species complex (FSAMSC). Among the ENNs, enniatin A (ENA), enniatin A1 (ENA1), enniatin B (ENB) and enniatin B1 (ENB1) analogues are commonly detected in cereals globally [57, 59–62]. Despite their widespread occurrence, ENNs are presently not classified as regulated mycotoxins. This is because the knowledge about their toxicity and their impact on human and animal health is still limited [56, 63–65]. The European Food Safety Authority (EFSA) highlighted concern for human and animal health regarding the possible interactions of ENNs with other mycotoxins and chronic exposure from contaminated food and feed [66]. In the *Fusarium* genus, ENN biosynthesis is catalysed by enniatin synthetase, a non-ribosomal multifunctional enzyme encoded by the *ESYN1* gene [67].

In comparison to DON, little is known about the effect of ENNs on plants or their role in fungal virulence. Early experiments in plants described an inhibition of germination and induction of wilting [68, 69]. Recent studies have demonstrated the induction of cell death, oxidative stress, as well as the reduction of shoot length in response to ENB [37, 70]. The role of ENNs in *F. avenaceum* virulence was preliminarily hypothesized [71] and subsequently confirmed in potato tubers [72]. By contrast, a relationship between ENNs and pathogenicity of *Fusarium oxysporum* f. sp. *melonis* on muskmelon was not detected [73]. Recently, the investigation on ENNs’ role in fungal virulence was expanded to other hosts, specifically durum wheat and pea, but again an impact of ENNs on *F. avenaceum* aggressiveness was only observed in potato tubers [74]. In contrast, a positive correlation between ENNs production, especially ENA1, and virulence was reported in barley seedlings and heads when comparing *F. avenaceum* isolates that naturally produce different levels of ENNs. This suggests that ENNs may indeed function as virulence factors in cereals [75].

Since ENN contamination of grains continues to be a problem in cereal grains around the world, a more comprehensive understanding of these compounds is needed. In this study, a *F. avenaceum* strain was compared to an ENN non-producing mutant (*ESYN1*-disrupted) and to an *ESYN1* overexpression mutant to elucidate the function of ENNs on roots, stem bases, and heads of common wheat. Additionally, the role of ENNs was compared with that of DON, a known virulence factor in wheat heads [32, 43, 76]. This involved testing the disease-causing capability of a trichothecene non-producing *F. graminearum* mutant (*TRI5*-disrupted strain developed in this study) against its DON-producing wildtype strain in roots, stem bases, and heads of common wheat.

## Results

### In vitro production of secondary metabolites by *Fusarium avenaceum* and *Fusarium graminearum* strains

Data on the in vitro production of secondary metabolites by the *F. avenaceum* strains grown on autoclaved rice are shown in Table 1 (ENNs) and in Table S1 (other detected secondary metabolites). The LC-MS/MS analysis confirmed that the *ESYN1*-disrupted strain (*Fa*Δ*esyn1*) did not produce ENNs, whereas the wild type (*Fa*WT) and the *ESYN1*-overexpressed strain (*FaESYN1*_OX) produced similar amounts of ENNs. Conversely, the other secondary metabolites were not significantly different between the three *F. avenaceum* strains, except for aurofusarin (Table S1).

Data on the in vitro production of secondary metabolites by the *F. graminearum* strains are shown in Table 2 (DON) and in Table S2 (other detected secondary metabolites). The *TRI5* knockout strain (*Fg*Δ*tri5*) was confirmed to be a non-producer of DON and its acetylated form 15-acetyl deoxynivalenol (15-ADON). Among the other secondary metabolites, no significant differences were found in aurofusarin and fusarin C accumulation between the wild type (*Fg*WT) and *Fg*Δ*tri5*. Conversely, *Fg*Δ*tri5* did not produce culmorins and produced significantly lower levels of butenolide, zearalenone, and zearalenone-sulfate in comparison with *Fg*WT.

**Table 1:** In vitro biosynthesis of enniatins (ng g^−1^) produced by *Fusarium avenaceum* strains *Fa*WT, *Fa*Δ*esyn1* and *FaESYN1*_OX as detected by LC-MS/MS in autoclaved rice

Secondary metabolites		*F. avenaceum* strain		
		*Fa*WT	*Fa*Δ*esyn1*	*FaESYN1*_OX
Enniatin A	ng g^−1^	1190	< LOD^c^	1130
	SE^a^	127	< LOD	129
	MCT^b^	a	b	a
Enniatin A1	ng g^−1^	27,500	< LOD	30,400
	SE	3300	< LOD	5150
	MCT	a	b	a
Enniatin B	ng g^−1^	265,000	< LOD	255,000
	SE	509	< LOD	6000
	MCT	a	b	a
Enniatin B1	ng g^−1^	178,000	< LOD	223,000
	SE	17,200	< LOD	32,000
	MCT	a	b	a
Enniatin B2	ng g^−1^	53,600	< LOD	73,200
	SE	6420	< LOD	11,800
	MCT	a	b	a
Enniatin B3	ng g^−1^	373	< LOD	439
	SE	57.7	< LOD	58.5
	MCT	a	b	a
Total enniatins	ng g^−1^	525,000	< LOD	583,000
	SE	27,400	< LOD	43,500
	MCT	a	b	a

^a^SE = ± Standard Error; ^b^MCT = Multiple Comparison Test; ^c^LOD = Limit of Detection

**Table 2:** In vitro biosynthesis of deoxynivalenol (ng g^−1^) produced by *Fusarium graminearum* strains *Fg*WT and *Fg*Δ*tri5* as detected by LC-MS/MS in autoclaved rice

Secondary metabolites		*F. graminearum* strains	
		*Fg*WT	*Fg*Δ*tri5*
15-Acetyldeoxynivalenol	ng g^−1^	332,000	< LOD^c^
	SE^a^	19,800	< LOD
	MCT^b^	a	b
Deoxynivalenol	ng g^−1^	138,000	< LOD
	SE	34,900	< LOD
	MCT	a	b
Total deoxynivalenol	ng g^−1^	470,000	< LOD
	SE	54,700	< LOD
	MCT	a	b

^a^SE = ± Standard Error; ^b^MCT = Multiple Comparison Test; ^c^LOD = Limit of Detection

### *Fusarium* virulence assays on different common wheat tissues

#### Fusarium seedling root assay

**Fig. 1:**
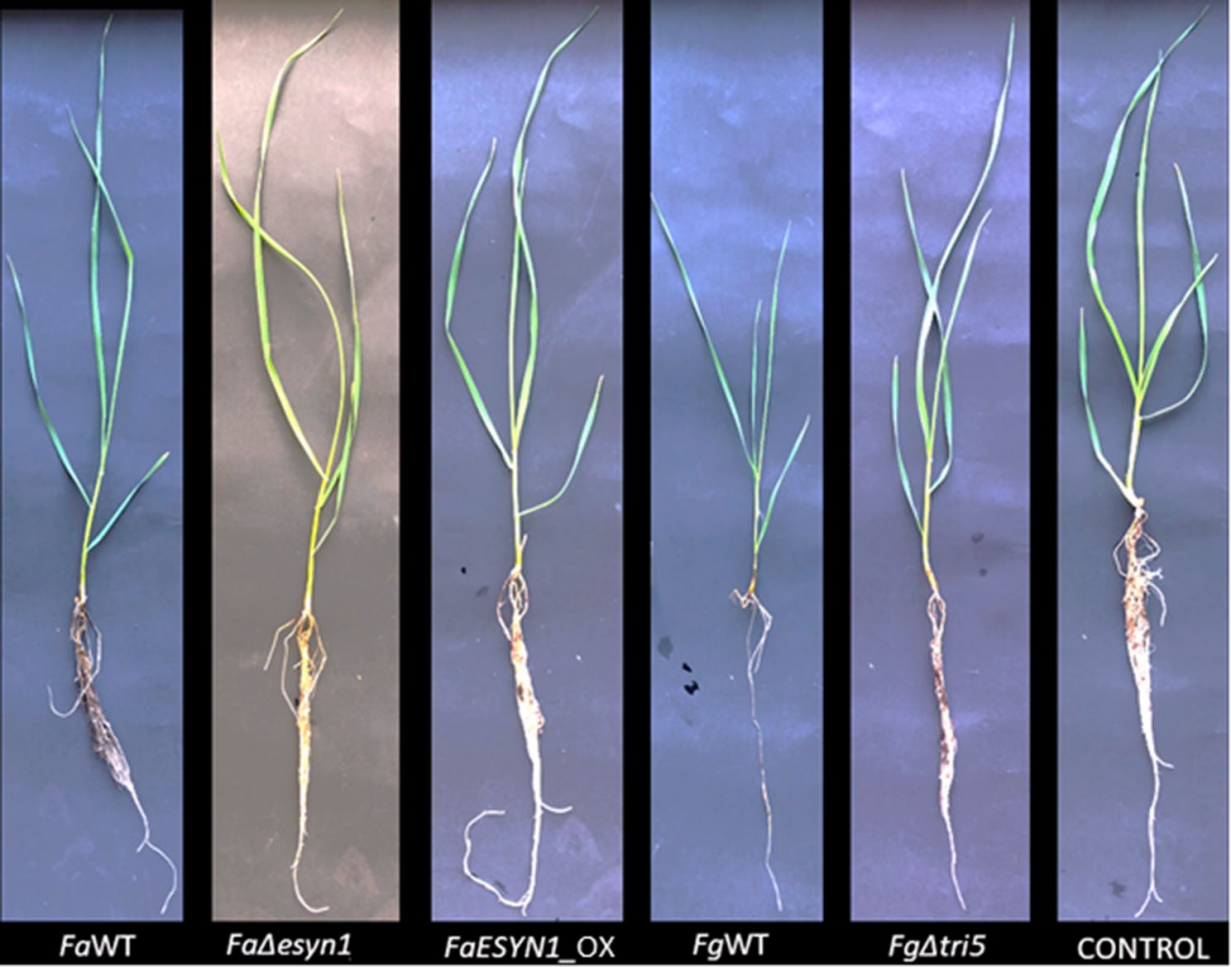
Development of seedlings of common wheat at 15 days post-inoculation of roots with *F. avenaceum* strains *Fa*WT, *Fa*Δ*esyn1* and *FaESYN1*_OX and *F. graminearum* strains *Fg*WT and *Fg*Δ*tri5*. The control was mock-inoculated with sterile water

**Fig. 2:**
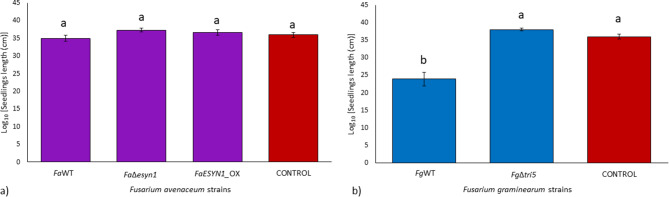
Length (cm) of common wheat seedlings (only the aerial part, excluding roots) at 15 days post-inoculation of the roots with (**a**) *F. avenaceum* strains *Fa*WT, *Fa*Δ*esyn1* and *FaESYN1*_OX and (**b**) *F. graminearum* strains *Fg*WT and *Fg*Δ*tri5*. The control was mock-inoculated with sterile water. Columns represent the log-transformed average (± Standard Error) of two independent experiments each composed of nine replicates (plants) for each fungal strain. Different letters (a-b) above the columns refer to significant differences (*p* < 0.05) according to Tukey’s honestly significant difference multiple comparison tests

Inoculation of seedling roots did not lead to significant growth reduction of the aerial part of the plants, as no difference in the average seedling length was observed between any of the fungal treatments and the control (Figs. 1 and 2), except for *Fg*WT, which resulted in a significant reduction (*p* < 0.05) of seedling length (Fig. 2b).

**Fig. 3:**
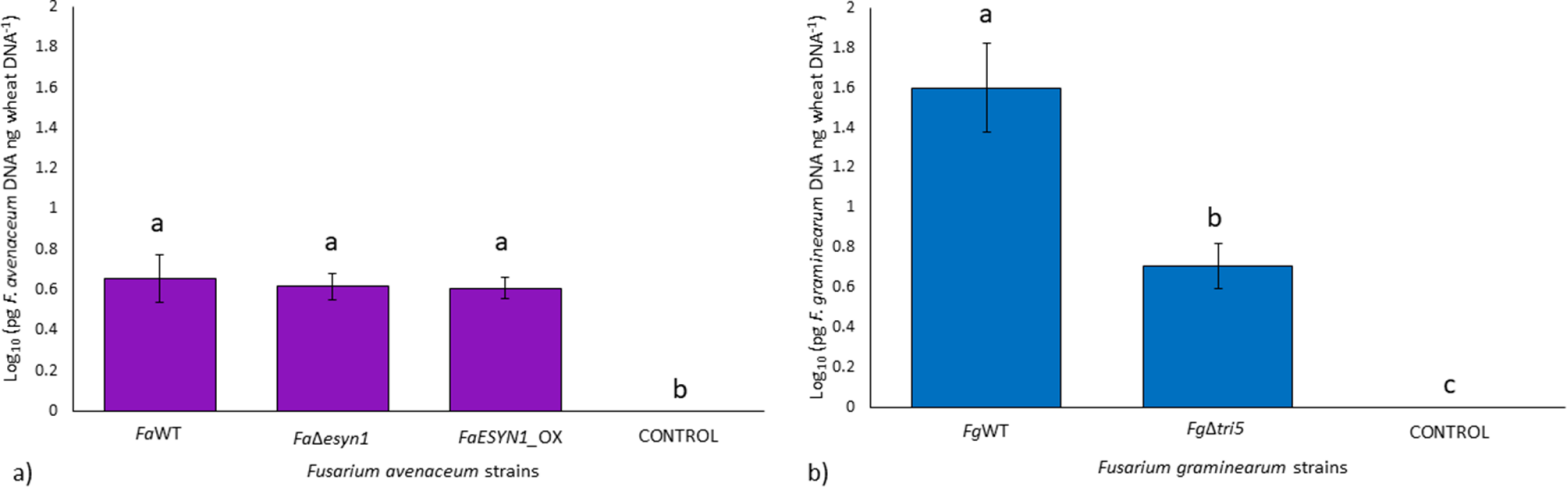
Fungal biomass accumulation in common wheat root tissues (pg DNA ng common wheat DNA^−1^) at 15 days post-inoculation with *F. avenaceum* strains *Fa*WT, *Fa*Δ*esyn1* and *FaESYN1*_OX (**a**) and *F. graminearum* strains *Fg*WT and *Fg*Δ*tri5* (**b**). The control was mock-inoculated with sterile water. The columns represent the log-transformed average (± Standard Error) of two independent experiments (each composed of three replicates, each composed of three roots bulked together). Different letters (a–c) above the columns refer to significant differences (*p* < 0.05) according to Tukey’s honestly significant difference multiple comparison tests

Fungal biomass accumulation in the roots was determined by DNA quantification of *F. avenaceum* and *F. graminearum* by qPCR. The R^2^ and the reaction efficiency calculated from the linear equation were 0.99 and 95% for *F. graminearum* and 0.98 and 98% for *F. avenaceum* (Fig. S1). The dissociation curve analysis showed specific amplification products in the presence of pure fungal DNA (standard curves) and *F. avenaceum* and *F. graminearum* DNA (samples). No target amplification was detected in the negative controls. Therefore, the Ct values used to quantify DNA were those for which the dissociation curve analysis showed the presence of specific amplification products. The reaction efficiency calculated from the common wheat roots linear equation was 97%, with R^2^ = 0.99 (Fig. S1). Roots inoculated with the three *F. avenaceum* strains did not show significant differences (*p* > 0.05) in their accumulation (Fig. 3a). Conversely, a significant difference was detected between the *F. graminearum* strains, where *Fg*WT accumulated higher amounts of *F. graminearum* DNA (*p* < 0.05) than *Fg*Δ*tri5* (Fig. 3b).

Together, these results show that ENN production by *F. avenaceum* did not affect seedling root rot occurrence in common wheat, but DON production by *F. graminearum* did increase disease severity and facilitated a higher accumulation of fungal biomass in the roots.

#### Fusarium seedling stem base assays

**Fig. 4:**
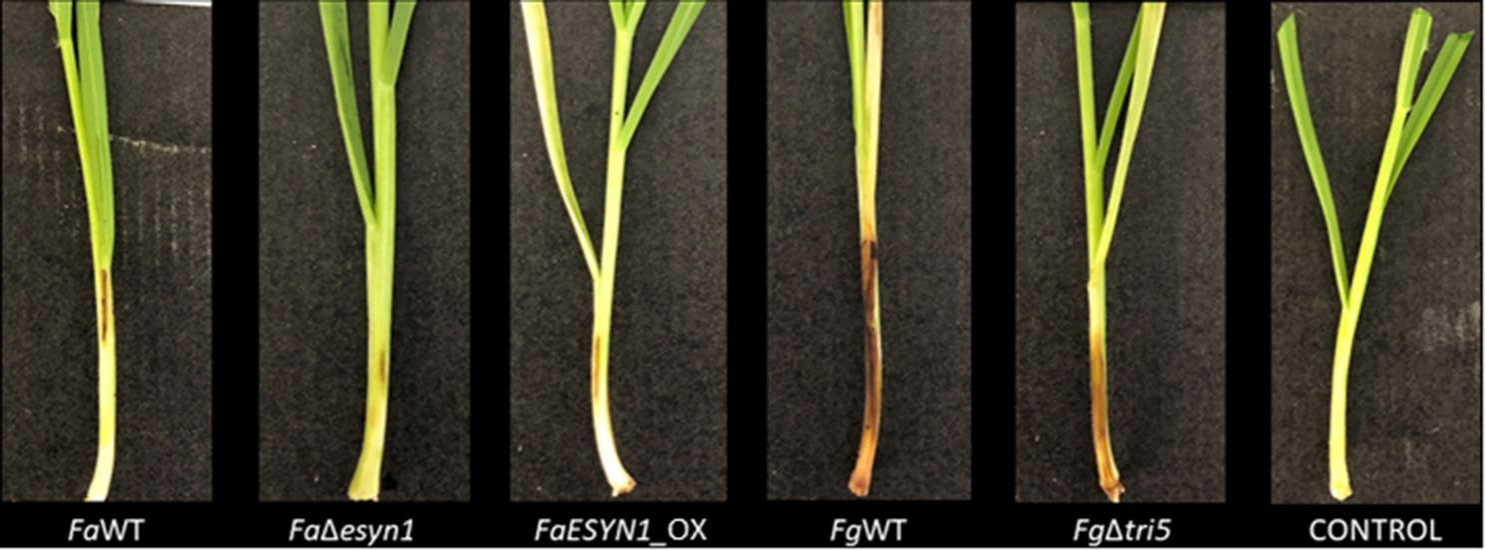
Symptoms developed on common wheat stem bases at 21 days post-inoculation with *F. avenaceum* strains *Fa*WT, *Fa*Δ*esyn1* and *FaESYN1*_OX and *F. graminearum* strains *Fg*WT and *Fg*Δ*tri5*. The control was mock-inoculated with PDA only

**Fig. 5:**
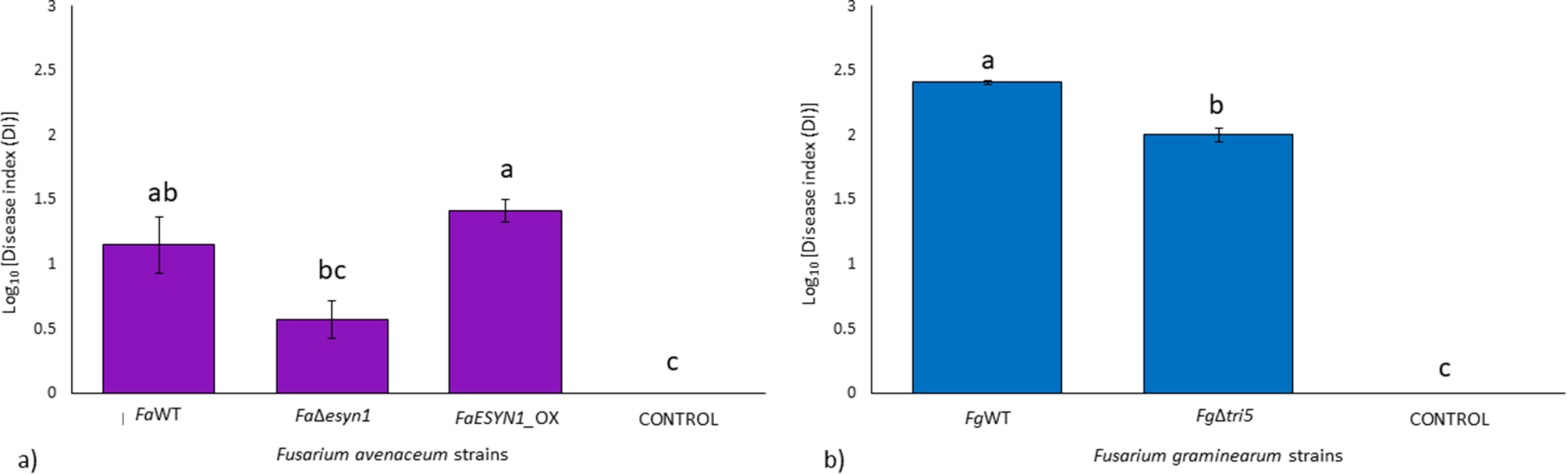
Disease Index (DI) assessed in stem base of common wheat seedlings at 15 days post-inoculation with (**a**) *F. avenaceum* strains *Fa*WT, *Fa*Δ*esyn1* and *FaESYN1*_OX and (**b**) *F. graminearum* strains *Fg*WT and *Fg*Δ*tri5*. The control was mock-inoculated with PDA only. Columns with bars represent the log-transformed average (± Standard Error) of two independent experiments each composed of 15 replicates (plants) for each fungal strain. The letters (a–c) above the columns refer to significant differences (*p* < 0.05) according to Tukey’s honestly significant difference multiple comparison tests

*F. avenaceum* strain *FaESYN1*_OX caused a significantly higher Disease Index (DI) (*p* < 0.05) in the wheat stem base when compared with that induced by *Fa*Δ*esyn1*; however, the DI induced by *Fa*WT was not significantly different from those of either mutant strain (Figs. 4 and 5a). A visual comparison of symptoms caused by *F. graminearum* strains compared to *F. avenaceum*, showed more necrosis in response to *F. graminearum* treatments (Fig. 4). A comparison of DI between the two *F. graminearum* strains shows that *Fg*WT caused significantly more disease (*p* < 0.05) than that induced by *Fg*Δ*tri5* (Figs. 4 and 5b).

**Fig. 6:**
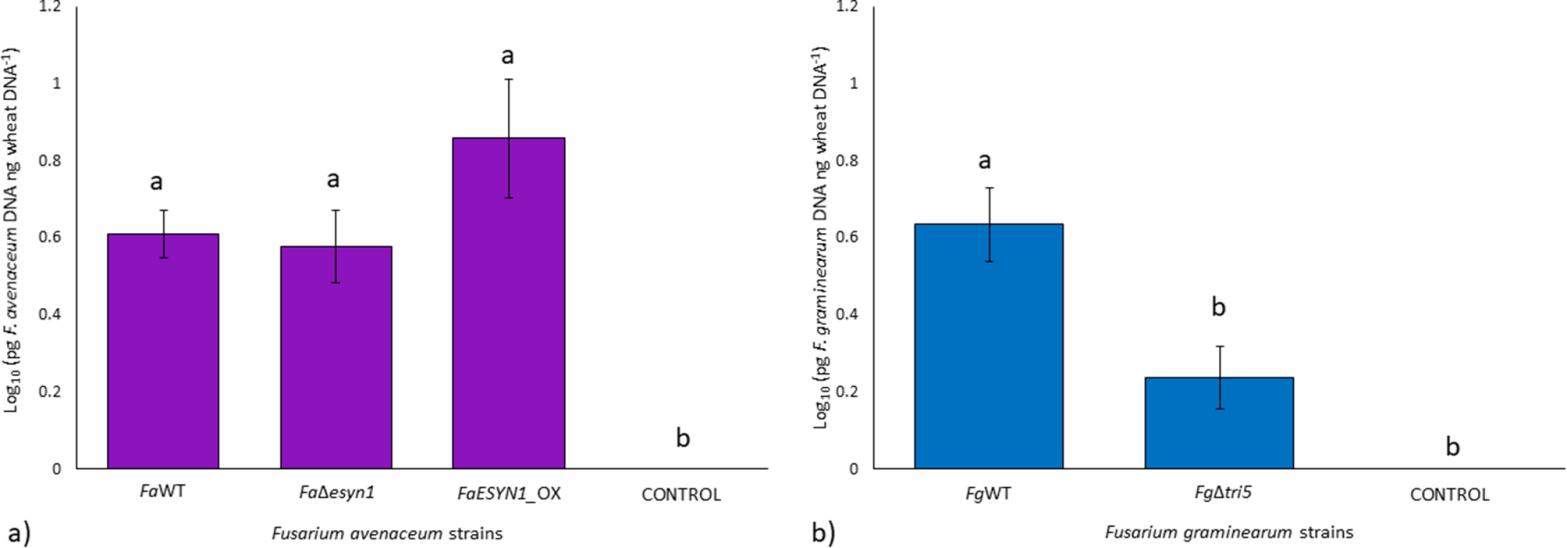
Fungal biomass accumulation in common wheat stem base tissues (pg DNA ng common wheat DNA^−1^) at 15 days post-inoculation with (**a**) *F. avenaceum* strains *Fa*WT, *Fa*Δ*esyn1*, and *FaESYN1*_OX and (**b**) *F. graminearum* strains *Fg*WT and *Fg*Δ*tri5*. The control was mock-inoculated with PDA. The columns represent the log-transformed average (± Standard Error) of two independent experiments (each composed of three replicates, each composed of five stem bases bulked together). Different letters (a-b) above the columns refer to significant differences (*p* < 0.05) according to Tukey’s honestly significant difference multiple comparison tests

The reaction efficiency of qPCR assays used for estimating fungal biomass accumulation in stem bases calculated from the *F. avenaceum* linear equation was 103% with R^2^ = 0.99, while for *F. graminearum* it was 104% with R^2^ = 0.99 (Fig. S2). The dissociation curve analysis showed specific amplification products and no target amplification was detected in the negative controls. The reaction efficiency calculated from the common wheat stem base linear equation was 104%, with R^2^ = 0.98 (Fig. S2). All three *F. avenaceum* strains showed a similar accumulation of fungal biomass (Fig. 6a), whereas, in the case of *F. graminearum* strains, *Fg*WT inoculations resulted in more biomass accumulation in stem bases (*p* < 0.05) than that detected for *Fg*Δ*tri5* (Fig. 6b).

Thus, the ability of *F. avenaceum* to produce ENNs did not appear to influence disease severity in the wheat stem base and did not affect fungal biomass accumulation in this tissue. By contrast, the ability of *F. graminearum* to produce DON had a significant positive impact on the disease in the stem base and also significantly increased the quantity of fungal accumulation in the same tissue.

#### Fusarium head assay

**Fig. 7:**
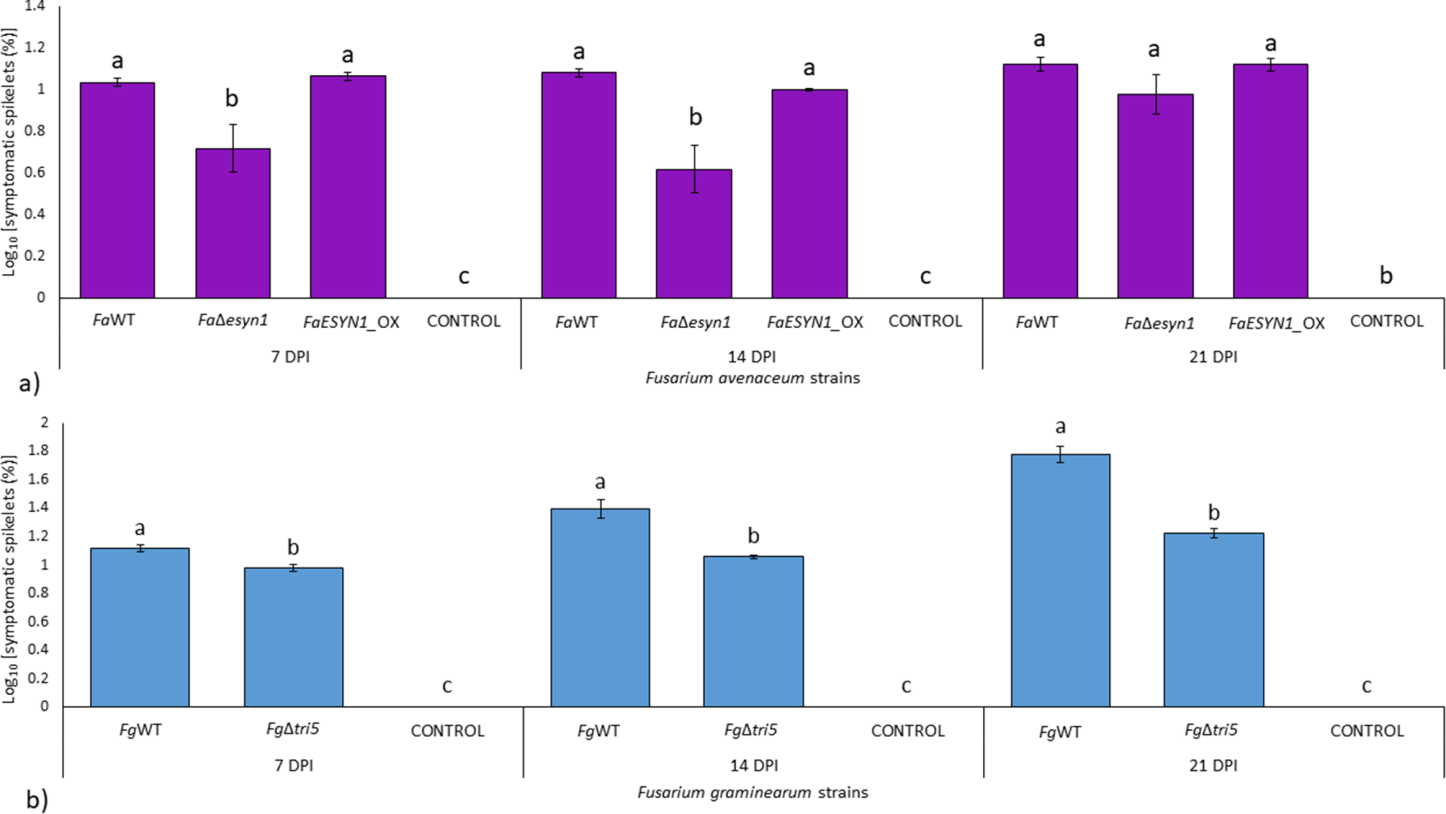
Fusarium head blight severity (%) on common wheat heads at 7, 14, and 21 days post-inoculation with *F. avenaceum* strains *Fa*WT, *Fa*Δ*esyn1* and *FaESYN1*_OX (**a**) and *F. graminearum* strains *Fg*WT and *Fg*Δ*tri5* (**b**). The control was mock-inoculated with sterile water. Columns with bars represent the log-transformed average (± Standard Error) of two independent experiments each composed of nine replicates (plants) for each fungal strain. The letters (a–c) above the columns indicate the presence of significant differences (*p* < 0.05) according to Tukey’s honestly significant difference multiple comparison tests

Results of FHB symptom observation are detailed in Table S3. In particular, wheat heads inoculated with *F. avenaceum* and *F. graminearum* strains resulted in the appearance of FHB symptoms at 7 days post-inoculation (dpi). In this case, *Fa*WT and *FaESYN1*_OX inoculations resulted in a small but significant increase in the percentage of symptomatic spikelets (*p* < 0.05) compared to *Fa*Δ*esyn1* (Fig. 7a). *Fg*WT also caused significantly higher levels (*p* <  0.05) of FHB symptoms than *Fg*Δ*tri5* (Fig. 7b).

**Fig. 8:**
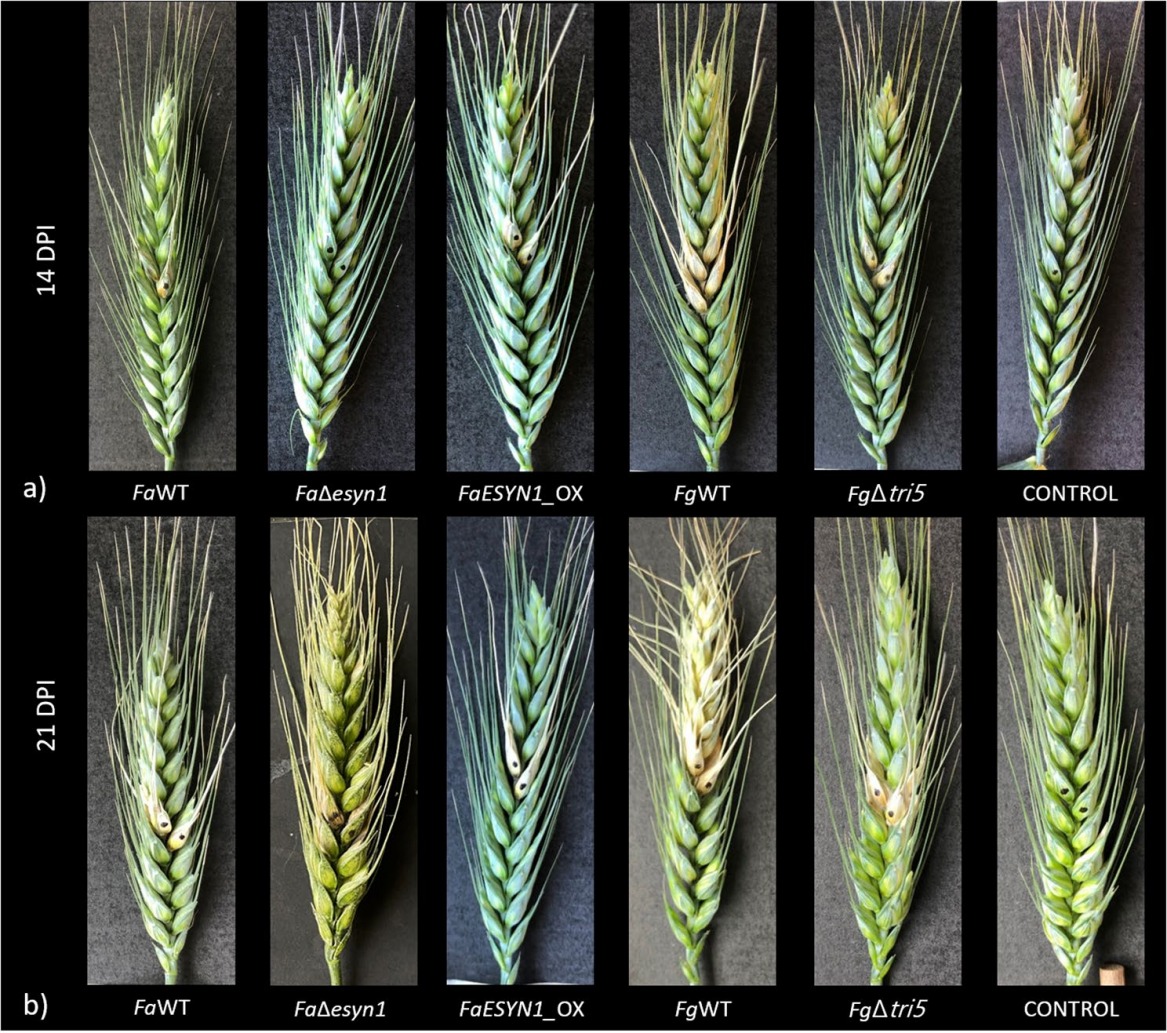
Fusarium head blight symptoms on common wheat heads at 14 (**a**) and 21 (**b**) days post-inoculation with *F. avenaceum* strains *Fa*WT, *Fa*Δ*esyn1* and *FaESYN1*_OX and *F. graminearum* strains *Fg*WT and *Fg*Δ*tri5*. The control was mock-inoculated with sterile water. Two central spikelets, marked with a black pen, were inoculated at mid-anthesis. A picture of one representative head per thesis was taken

The same trend described at 7 dpi, was observed also at 14 dpi (Figs. 7 and 8) where, again, the strains unable to produce ENNs (*Fa*Δ*esyn1*) or DON (*Fg*Δ*tri5*) caused a significantly lower percentage of symptomatic spikelets (*p* < 0.05) than that detected in the heads inoculated with strains able to produce ENNs (*Fa*WT and *FaESYN1*_OX) or DON (*Fg*WT), respectively.

FHB symptoms at 21 dpi were present at a lower level on the heads inoculated with *Fa*Δ*esyn1* (Fig. 7), but they were not significantly different (*p* < 0.05) in the heads inoculated with the three *F. avenaceum* strains (Figs. 7 and 8). By contrast, *F. graminearum Fg*WT was able to maintain a higher level of severity (*p* < 0.05) than that detected in *Fg*Δ*tri5*.

**Fig. 9:**
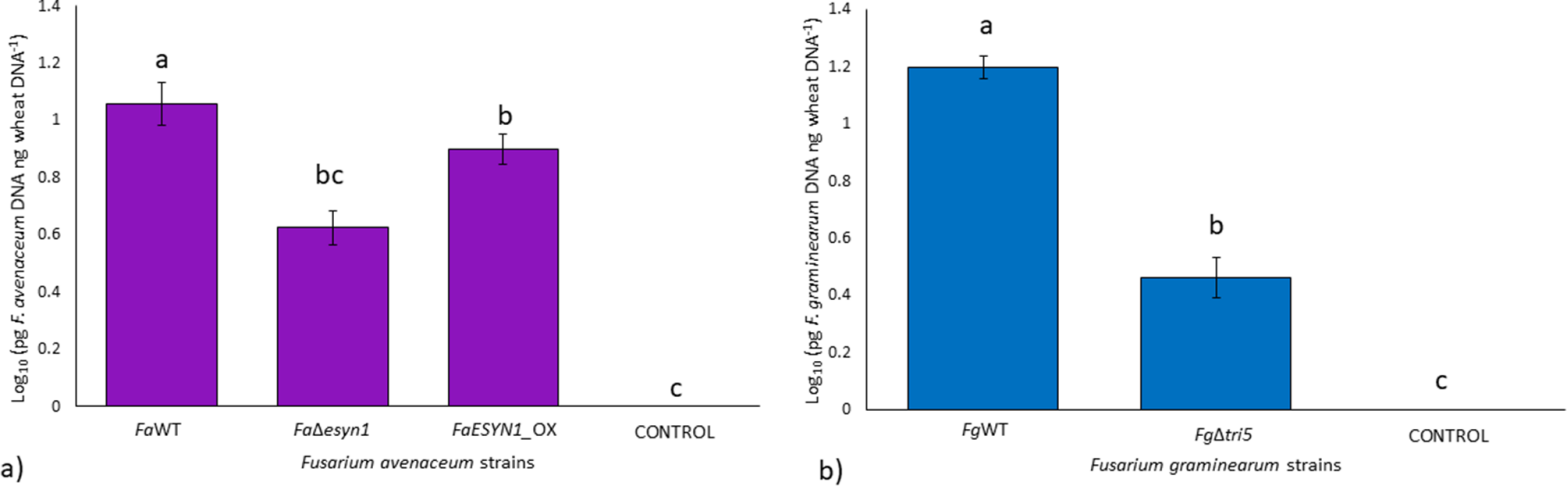
Fungal biomass accumulation in common wheat heads (pg DNA ng common wheat DNA^−1^) at 28 days post-inoculation with (**a**) *F. avenaceum* strains *Fa*WT, *Fa*Δ*esyn1*, and *FaESYN1*_OX and (**b**) *F. graminearum* strains *Fg*WT and *Fg*Δ*tri5*. The control was mock-inoculated with PDA. The columns represent the log-transformed average (± Standard Error) of two independent experiments (each composed of three replicates, in turn, composed of three heads bulked together). Different letters (a-b) above the columns refer to significant differences (*p* < 0.05) according to Tukey’s honestly significant difference multiple comparison tests

qPCR assays were used to estimate fungal biomass accumulated in the head at 28 dpi (Fig. 9). R^2^ values calculated from the linear equations of the three standard curves were 0.99 for *F. graminearum*, *F. avenaceum*, and common wheat head. Reaction efficiencies obtained from the linear equations of the three standard curves were 104% for common wheat head and *F. graminearum,* and 103% for *F. avenaceum*. The dissociation curve analysis showed specific amplification products in the presence of pure fungal DNA (standard curves) and the presence of DNA of the two *Fusarium* species analyzed (samples). No target amplification was detected in negative controls (Fig. S3).

The two species analysed by qPCR were detectable through the presence of DNA accumulation on heads harvested at 28 dpi. Focusing on *F. avenaceum* strains (Fig. 9a), *Fa*WT accumulated in the head tissue in higher abundance than *Fa*Δ*esyn1*, where significantly higher biomass (*p* < 0.05) was observed in the *Fa*WT inoculated heads. Meanwhile, the biomass of *FaESYN1_*OX did not differ significantly (*p* < 0.05) from either the *Fa*WT or *Fa*Δ*esyn1*. Regarding *F. graminearum* strains (Fig. 9b), *Fg*WT showed a significantly higher amount of biomass (*p* < 0.05) in comparison to *Fg*Δ*tri5*.

In summary, ENN production by *F. avenaceum* increased disease symptoms, and while the difference in the percentage of diseased spikelets was no longer significant by 21 dpi, the amount of fungal biomass accumulation remained lower in the heads inoculated with *Fa*Δ*esyn1* when compared to the *Fa*WT but not when compared with *FaESYN1*_OX by 28 dpi.

The amount of ENNs detected in common wheat heads at 28 dpi with the three *F. avenaceum* strains is shown in Table 3. As expected, no ENNs were detected in the heads inoculated with *Fa*Δ*esyn1*, but they did accumulate in the heads inoculated with either *Fa*WT and *FaESYN1*_OX (Table S4). Accumulation of aurofusarin and chrysogin in inoculated heads was the same among the three *F. avenaceum* strains (*p* > 0.05). Moniliformin also accumulated to a similar amount (*p* > 0.05) in both *Fa*WT and *FaESYN1*_OX, showing significantly higher levels (*p* < 0.05) compared to *Fa*Δ*esyn1*.

**Table 3:** Enniatins (ng g^−1^) detected in common wheat heads at 28 days post-inoculation following head inoculation with *F. avenaceum* strains

Secondary metabolites		*F. avenaceum* strain		
		*Fa*WT	*Fa*Δ*esyn1*	*FaESYN1*_OX
Enniatin A	ng g^−1^^a^	30.6	< LOD^d^	7.85
	SE^b^	5.88	< LOD	1.73
	MCT^c^	a	b	b
Enniatin A1	ng g^−1^	595	< LOD	164
	SE	131	< LOD	32.3
	MCT	a	b	b
Enniatin B	ng g^−1^	3140	< LOD	2200
	SE	533	< LOD	509
	MCT	a	b	a
Enniatin B1	ng g^−1^	2300	< LOD	936
	SE	323	< LOD	176
	MCT	a	c	b
Total enniatins	ng g^−1^	6060	< LOD	3310
	SE	993	< LOD	719
	MCT	a	c	b

^a^average of two independent experiments (each composed of three replicates, each composed of three heads bulked together); ^b^SE = ± Standard Error; ^c^MCT = Multiple Comparison Test; ^d^LOD = Limit of Detection

In the head tissue inoculated with *F. graminearum* strain *Fg*Δ*tri5*, the trichothecene DON and its acetylated derivates were not detected (Table 4), and, similarly, DON-3-glucoside was absent (Table S5). Meanwhile, the head tissues inoculated with *Fg*WT resulted in DON and acetylated-DON (3-ADON and 15-ADON) accumulation (Table 4). Among the other secondary metabolites, similar levels (*p* > 0.05) of aurofusarin and chrysogin were detected in the heads inoculated with either *Fg*WT and *Fg*Δ*tri5*. Finally, 15-hydroxyculmorin, 5-hydroxyculmorin, 15-hydroxyculmorun, culmorin and butenolide were mainly produced (*p* < 0.05) by *Fg*WT (Table S5).

**Table 4:** Deoxynivalenol and its acetylated forms (ng g^−1^) detected in common wheat heads at 28 days post-inoculation following head inoculation with *F. graminearum* strains

Secondary metabolites		*F. graminearum* strains	
		*Fg*WT	*Fg*Δ*tri5*
3-Acetyldeoxynivalenol	ng g^−1^^a^	308	< LOD^d^
	SE^b^	121	< LOD
	MCT^c^	a	b
15-Acetyldeoxynivalenol	ng g^−1^	2490	< LOD
	SE	1170	< LOD
	MCT	a	b
Deoxynivalenol	ng g^−1^	29,280	< LOD
	SE	5390	< LOD
	MCT	a	b
Total deoxynivalenol	ng g^−1^	32,100	< LOD
	SE	6680	< LOD
	MCT	a	b

^a^average of two independent experiments (each composed of three replicates, each composed of three heads bulked together); ^b^SE = ± Standard error; ^c^MCT = Multiple Comparison Test; ^d^LOD = Limit of Detection

## Discussion

FHB is a disease affecting wheat around the world. The most challenging problem associated with this disease is mycotoxin accumulation that occurs in grains of infected heads and that compromises the quality and safety of raw materials, as well as processed food and feed, for human and animal consumption [77]. Mycotoxins are secondary metabolites and are therefore not required for the growth and development of fungi; however, these molecules can influence the fitness of the producing organism in some environments. For example, certain mycotoxins, such as DON, have been shown to enhance fungal virulence [78, 79], as observed for FHB in the *F. graminearum*-wheat interaction [76]. The significance of DON was initially demonstrated by generating and studying a trichothecene non-producing mutant strain, with several subsequent studies reinforcing these findings [32]. In contrast, despite their frequent occurrence, little is known about other *Fusarium* secondary metabolites such as ENNs [80]. For this reason, in the present study, the role of ENNs in *F. avenaceum* virulence was investigated on three different common wheat tissues and compared to that of DON in *F. graminearum*.

This study highlights the importance of DON in *F. graminearum* virulence in common wheat, regardless of the infected tissue. In wheat roots, the DON-producing *F. graminearum* wild-type strain (*Fg*WT) stunted seedling development and accumulated in higher abundance than in roots inoculated with the DON non-producing mutant (*Fg*Δ*tri5*) (Figs. 2b and 3b). The involvement of DON in *F. graminearum* virulence in seedling blight and root rot has previously been demonstrated in wheat, barley and triticale [81]. Conversely, it has been demonstrated that the capability of *F. graminearum* and *F. culmorum* to produce trichothecenes adversely affects the early stages of wheat root colonization [82]. This suggests the hypothesis that tissues lacking pigments, such as roots, might be less affected by the trichothecene mycotoxins than green tissues. The different outcomes reported among these studies might be explained by differences in cultivars or methodologies and demonstrate the need for further investigation on DON virulence in tissues other than the wheat head.

The present study also showed that DON acts as a virulence factor for *F. graminearum* in the seedling stem base of common wheat. In fact, DON-producing *Fg*WT provoked higher symptom severity and resulted in a higher accumulation of fungal biomass in the seedling stem base when compared to the DON non-producing strain, *Fg*Δ*tri5* (Figs. 5b and 6b). These findings are consistent with previous reports in which DON acts as a virulence factor in stem base infection of wheat [76, 82, 83]. Interestingly, in one study investigating Fusarium crown rot, DON production in *F. graminearum* did not contribute to virulence in the wheat stem base, but did contribute to stem colonization [84].

Similarly to the results observed in this study in roots and stem bases of wheat seedlings, an increased virulence was observed in wheat heads infected with the DON-producing *F. graminearum* strain. A higher degree of disease symptom severity was observed, coupled with a higher accumulation of fungal biomass, in the wheat heads infected with *Fg*WT compared to *Fg*Δ*tri5* (Figs. 7b and 9b). The role of DON as a virulence factor in FHB disease has been described in detail in previous studies [32, 43, 45, 76, 85]. Restricted growth of a *TRI5*-disrupted mutant, where the fungus was retained in the infected spikelets, was observed [43], indicating that DON may facilitate the spread of *F. graminearum* throughout the wheat head. Results of the present research are in alignment with previous results, as disease spread was not observed in *Fg*Δ*tri5* infected heads of common wheat.

Interestingly, the role of trichothecene mycotoxins in virulence is host- and chemotype-specific [86], where chemotype refers to the chemical profile, in this case the trichothecene profile, of the fungus [87]. For example, while DON non-producing (*TRI5-*disrupted) mutants have reduced virulence in wheat heads, the same is not true for NIV non-producing mutants compared with their NIV producing wild-type strains [86], which are generally less aggressive than DON chemotypes [4]. By contrast, NIV appears to contribute to virulence in maize [86]. Furthermore, *TRI5*-disruption of DON-producing strains does not affect disease spread in barley heads as it does in wheat [86].

ENNs are a different class of mycotoxins than trichothecenes and have a different mode of action which is thought to be via their interaction with cell membranes and subsequent creation of cation-selective pores [65]. Their role in *F. avenaceum* virulence in common wheat appears to be less straightforward than that of the DON in the *F. graminearum*-wheat interaction. Using an *ESYN1*-disrupted strain (*Fa*Δ*esyn1*) and an *ESYN1*-overexpressed strain (*FaESYN1*_OX) of *F. avenaceum* (*Fa*WT), the role of ENNs was investigated in the same three tissues comparing their effects to those of DON.

Generally, the results indicate that ENNs are not crucial for *F. avenaceum* virulence in common wheat, but a possible tissue-dependent interaction was observed. ENNs did not act as a virulence factor for *F. avenaceum* in common wheat roots, as observed in our disease assay and also as shown by fungal biomass accumulation (Figs. 2a and 3a). Similarly, in a previous study with the same *F. avenaceum* strains (WT and mutants) but on pea roots, ENNs did not appear to influence *F. avenaceum* virulence in this host/tissue [74]. Using these same strains, ENNs were shown to affect *F. avenaceum* virulence in potato tubers [74], confirming the previously reported role of ENN production, and influencing virulence in potato tuber necrosis [71, 72]. Given *F. avenaceum* polyphagia, further studies on other hosts are necessary to test and better define the role of ENNs on virulence in roots and other hypogeal organs.

Similarly to what was observed for wheat roots, no differences were observed among the *F. avenaceum* isolates, *Fa*WT, *Fa*Δ*esyn1*, and *FaESYN1*_OX, in terms of virulence or biomass accumulation during stem base infection of common wheat seedlings (Figs. 5a and 6a). These results are consistent with earlier studies suggesting that ENNs do not appear to contribute to virulence in wheat and rye seedlings [88]. Conversely, in a recent study, a correlation was reported between the virulence of *F. avenaceum* and ENA1 production in planta [75]. In that study, the authors inoculated malting barley plants by growing seeds in soil amended with macerated mycelium and compared the effect of nine *F. avenaceum* isolates, all of which produced ENN, but those producing ENA1 in planta were the most aggressive in their ability to cause seedling blight. For this reason, the authors proposed ENNs, and in particular ENA1, as potential virulence factors for *F. avenaceum* in cereals. Based on these observations, the role of ENNs in *F. avenaceum* virulence in the stem base of cereal seedlings is something to be further investigated. In fact, given the lower virulence of *Fa*Δ*esyn1* in comparison to *FaESYN1*_OX in the present study, but not to *FaWT*, it would be interesting to determine whether there might be any differences in ENN production between the two ENN-producing strains in their interaction with the stem base of common wheat, and, if so, this might suggest a quantitative effect of these compounds in virulence. While in our work an increase in ENN accumulation was not observed for the *FaESYN1*_OX strain grown in culture, this strain was previously found to show higher ENN accumulation in both axenic cultures and in pea roots [74]. These results demonstrate that ENN production can be higher in the *FaESYN1*_OX strain under certain conditions and/or host interactions, although it is not clear whether such a difference may occur in the wheat stem base.

In the present work, a possible contribution of ENNs to fungal virulence was observed in the wheat head. Inoculation with *Fa*Δ*esyn1* resulted in a reduction of FHB symptoms at 7 and 14 dpi, compared to inoculation with either of the ENN-producing strains (*Fa*WT and *FaESYN1_*OX). This effect was no longer significant at 21 dpi (Fig. 7a), suggesting that the lack of ENN production can cause an initial delay in FHB symptoms. Importantly, while the percentage of diseased spikelets (considered here as disease severity) is similar among the three treatments at 21 dpi, the visual symptoms observed in the diseased spikelets appear to be less severe (Fig. 8). This result is partially supported by the lower fungal biomass accumulation of the *Fa*Δ*esyn1* in comparison to *Fa*WT observed at 28 dpi (Fig. 9a). While, a slight difference, even if not significant, was observed between *Fa*Δ*esyn1* and *FaESYN1_*OX fungal biomass.

It should be noted that the role of ENNs for *F. avenaceum* in FHB virulence is less impactful than the role of DON for *F. graminearum.* In fact, the reduction in biomass accumulation in the absence of DON (*FgWT* vs. *Fg*Δ*tri5*) is around 60%, whereas in the absence of ENNs (*FaWT* vs. *Fa*Δ*esyn1*) is around 40%.

*F. graminearum* is itself more aggressive than *F. avenaceum*, and will spread easily from spikelet to spikelet, except in the most highly resistant cultivars [89, 90]. In the absence of trichothecene production in *F. graminearum*, the fungus is restricted to the inoculated spikelet of wheat. In the case of *F. avenaceum*, point inoculation of an individual spikelet in common wheat rarely results in disease spread from the inoculated spikelet (Table S3; [74]). As such, the effect of ENN disruption is less evident than that of DON. The same isolates were previously used to evaluate the role of ENNs in virulence in durum wheat, where disease spread frequently occurs, but no difference was observed in disease severity among the *ESYN1*-disruption and ENN-producing strains [74]. In durum wheat, biomass accumulation was not evaluated and it is unknown whether the difference in the percentage of diseased spikelets per head was representative of biomass accumulation within the head [74]. In the present work, a delay in the onset of symptoms was observed in response to *Fa*Δ*esyn1* in common wheat, as recorded at 7 and 14 days. In the previous work [74], disease spread was already observed at 14 dpi in durum wheat and it would have been interesting to determine whether an earlier evaluation date in durum wheat would have resulted in a different outcome. In barley, it was shown that, similarly to what the same authors observed in barley seedlings (described above), *F. avenaceum* isolates that produced ENA1 in planta caused more FHB disease than those that did not, suggesting that ENA1 might be an important player in *F. avenaceum* virulence [75]. Taken together, the results indicate that ENNs could contribute to virulence in cereals, though the effect is not as prominent or widespread as the role of DON.

In this study, ENNs appeared to confer a specific advantage only on a particular tissue of common wheat. Transcriptomic analysis of *Brachypodium distachyon* in response to infection of head and root tissues by *F. graminearum* exhibits many tissue-specific differences and among these, some are related to phytohormones, or the expression of genes associated with ROS and antioxidant production [91]. While in the case of common wheat DON appears to contribute to virulence regardless of tissue type, a different defense response mounted by different tissues within a host plant might readily explain differences in resistance to infection of those tissues. ENNs play an important role in ROS production [65] and could also influence hormonal defence pathways. Preliminary results from our group indicate that ENNs, in particular ENB, may also affect the expression of salicylate and jasmonate-related genes in a concentration-dependent manner (unpublished data). It would be interesting to determine whether ENN accumulation differs in different wheat tissues during *F. avenaceum* infection, and if so, whether this may result in different host-responses, such as those regulated by plant hormones.

## Conclusions

The present study confirms the role of DON as a crucial virulence factor for *F. graminearum* infection of common wheat, regardless of the infected tissue, whereas the role of ENNs in *F. avenaceum* virulence was reduced compared to DON and appeared to be tissue-dependent. Our results suggest that ENNs, in addition to being able to confer an advantage to *F. avenaceum* only on particular hosts [71, 72, 74, 75, 88], could also confer an advantage only on particular tissues in a given host. It is currently unknown what factor is responsible for the tissue-specific role of ENNs in *F. avenaceum* and why ENNs appear only to affect virulence in wheat heads and not in seedling roots and stem bases.

## Materials and methods

### Fungal material

To explore the role of ENNs in fungal virulence on the three different tissues of common wheat, *F. avenaceum* DAOM242378 (*Fa*LH27), its ENN non-producing mutant (*ESYN1*-disrupted) *Fa*LH27Δ*esyn*1_8, and *in locus* ENN over-expression mutant *Fa*LH27*ESYN1*_OX6, previously developed [74], were used. The *F. avenaceum* strains are described here in this paper as *Fa*WT (wild type), *FaESYN1*_OX (*ESYN1-*overexpressed), and *Fa*Δ*esyn1* (*ESYN1*-disrupted) (Table 5).

To compare the role of ENNs in virulence with that of DON, a *F. graminearum* wild-type strain and its *TRI5* deletion mutant were investigated. The *F. graminearum TRI5*-disruption (AB123 described here as *Fg*Δ*tri5*) was created in the wild-type CS3005 background (*Fg*WT) (Table 5). The *Fg*Δ*tri5* strain was generated via protoplast-mediated transformation of *F. graminearum* isolate CS3005, as previously described [41], using a synthesised DNA fragment ordered from GenScript (Piscataway, NJ, USA) that contained 1000 bp of sequence immediately upstream of the *TRI5* start codon, a short barcoding sequence (Table S6), an *Aspergillus nidulans TRPC* promoter nourseothricin acetyl transferase cassette corresponding to nucleotide positions 437–1387 of GenBank accession AY631958.2, and 993 bp of sequence immediately downstream of the *TRI5* stop codon. Transformants were selected on 50 mg L^− 1^ nourseothricin sulfate (Werner Bioagents, Jena, Thuringia, Germany). Transformants were screened for successful knockout of the *TRI5* gene using a three-primer PCR reaction (Table S6) designed such that the presence of the transforming DNA and absence of the DNA deleted from the genome was confirmed in a single PCR reaction as previously described [92, 93].

**Table 5:** Description of *Fusarium* strains used in this study

Strain description	Species	Source nomenclature	Reference
*Fa*WT	*F. avenaceum*	DAOM242378 (*Fa*LH27)	[94]
*FaESYN1*_OX	*F. avenaceum*	*Fa*LH27*ESYN1*_OX*6*	[74]
*Fa*Δ*esyn1*	*F. avenaceum*	*Fa*LH27Δ*esyn1_8*	[74]
*Fg*WT	*F. graminearum*	CS3005	[95]
*Fg*Δ*tri5*	*F. graminearum*	AB123	This study

### Secondary metabolite production by *F. avenaceum* and *F. graminearum* strains in rice cultures

To characterize the in vitro secondary metabolite production of the *F. avenaceum* and *F. graminearum* strains used in the present work, the protocol previously described was followed [16]. Briefly, 20 g of rice were added to 10 mL of deionized water in 100-mL glass flasks and autoclaved twice at 24 h intervals. Autoclaved flasks were inoculated with mycelial plugs (0.5 cm in diameter) taken from pure cultures of *Fa*WT, *Fa*Δ*esyn1*, *FaESYN1*_OX, *Fg*WT, and *Fg*Δ*tri5* grown for one week on potato dextrose agar (PDA, Biolife Italiana, Milan, Italy) at 22°C in the dark. Control flasks were inoculated with PDA plugs from clean plates without fungus. Once inoculated, flasks were incubated for 4 weeks in the dark at 22°C. The fungal cultures were subsequently placed into 50-mL plastic tubes (Thermo Fisher Scientific, Waltham, MA, USA) and, after 2 days at −80°C, were freeze-dried with a Heto Powder Dry LL3000 (Thermo Fisher Scientific) lyophilizer, finely ground with a Mixer Mill 400 (Retsch, Haan, Germany) for 6 min at 25 Hz, and stored at −80°C.

Five grams of each milled sample was used for secondary metabolite extraction and quantification as previously described [96]. Extraction was performed in 50-mL polypropylene tubes (Sarstedt, Nümbrecht, Germany) using 20 mL of extraction solvent (acetonitrile/water/acetic acid 79:20:1, v/v/v). After 90 min of shaking on a GFL 3017 rotary shaker (GFL, Burgwedel, Germany), 500 µL of the raw extract were transferred to vials and diluted with an equal volume of the dilution solvent (acetonitrile/water/acetic acid 20:79:1, v/v/v). In cases where the metabolite concentration exceeds the highest calibration standard, extracts were diluted 1:50 and/or 1:1000 (v/v), and re-analyzed.

Metabolite detection and quantification was performed using a qTrap 5500 MS/MS system (Sciex, Foster City, CA, USA) equipped with a TurboV electrospray ionisation (ESI) source, coupled to a 1290 series UHPLC system (Agilent Technologies, Waldbronn, Germany). Chromatographic separation was performed at 25°C on a Gemini C18-column, 150 × 4.6 mm i.d., 5 μm particle size, equipped with a C18 security guard cartridge, 4 × 3 mm i.d. (both Phenomenex, Torrance, CA, USA). Two MS/MS transitions were acquired per analyte except for moniliformin and 3-nitroropionic acid that each yield only one product ion. For confirmation of identification, the ion ratio had to agree with the relative values of the related authentic standard within 30% and an in-house retention time criterion of ± 0.03 min was applied. Quantification was based on external calibration using a serial dilution of a multi-analyte stock solution. The accuracy of the method is verified on a routine basis by participation in a proficiency testing scheme organized by BIPEA (Gennevilliers, France) with a rate of satisfactory z-scores of −2 < z < 2 of 96% for the > 2000 results submitted to date. LODs and LOQs for DON and ENNs are reported in Table S7.

### Plant growth conditions

Briefly, common wheat seeds (cv. A416, with known susceptibility to FHB) were surface sterilized in a water: sodium hypochlorite (7%; Carlo Erba Reagents, Milan, Italy) sterile solution [90:10 (v/v)] and rinsed three times with sterile deionized water. Seeds were placed in 14-cm Petri dishes (Nuova Aptaca, Asti, Italy) containing three filter papers soaked with 15 mL of sterile water. To promote the germination process, the Petri dishes were incubated in the dark at 4°C for 3 days and then at 22°C for 2 days. The germinated seeds were transplanted either into 24-well plates (Dominique Duthscher Group, Bernolsheim, France) in water-agar (1% w/v; Biolife) or pots filled with “traysubstrat” soil (Klasmann-Deilmann GmbH, Geeste, Germany). Plants grown in plates or pots were reared in a Conviron^®^ growth chamber (Controlled Environments Limited, Winnipeg, Manitoba, Canada) at 22°C and 80% relative humidity, with a 16 h photoperiod (light intensity starting at 10% and increasing hourly by 10% for 4 h where it was maintained at 50% for 8 h and then was decreased hourly in 10% increments until 16 h), or in a greenhouse at the Department of Agricultural, Food and Environmental Sciences (University of Perugia, Perugia, Italy) at 22 ± 4°C and 12 h photoperiod (supplied with artificial light in seasons when natural light was not sufficient) as specified and described in the virulence assay sections.

### *Fusarium* virulence assays on different tissues of common wheat

To investigate the role of ENNs and DON on fungal virulence, three assays were carried out on common wheat (cv. A416) to compare the virulence of *F. avenaceum* and *F. graminearum* wild types and mutant strains on three different host tissues: seedling roots, seedling stem bases, and heads.

#### *Fusarium* seedling root assay

*F. avenaceum* strains (*Fa*WT, *Fa*Δ*esyn1*, and *FaESYN1*_OX) and *F. graminearum* strains (*Fg*WT and *Fg*Δ*tri5*) were cultured in the dark at 22°C for 7 days in 9-cm Petri dishes with PDA for producing mycelium. Mung bean broth was prepared by adding 40 g of mung bean to 1 L of sterile water and boiling for 10 min. The beans were then separated from the broth through filtration with cheesecloth and the broth was autoclaved. One liter of mung bean broth in 2-L flasks was inoculated with a mycelial plug of the different strains and aerated with forced sterile air for 5 days at room temperature. All resulting conidial suspensions were filtered through Miracloth (EMD Millipore Corporation, Billerica, MA, USA) and the conidia were collected by centrifugation at 3000 rpm for 15 min at 4°C in a 5804 R centrifuge (Eppendorf, Hamburg, Germany). Finally, the concentration of conidia suspension was adjusted to 5 × 10^6^ macroconidia mL^−1^ using a hemocytometer.

The seedling root assay was based on a previously described method [97], with some modifications. Common wheat seedlings were grown in 24-well plates and incubated in the growth chamber. At the development stage first leaf unfolded (BBCH11) [98], the seedlings were moved from water-agar, wrapped at stem level with aluminium foil and placed in 50-mL tubes (Thermo Fisher Scientific), immersing only the roots in 10 mL of conidial suspension (1 × 10^6^ macroconidia mL^−1^) of the different strains. The roots of the control seedling were placed in distilled sterile water only. Tubes were placed at 180 rpm on a rotary shaker (Lab-line Instruments, Melrose Park, IL, USA) at room temperature for 24 h. Afterwards, the seedlings were individually transplanted into 5 × 5 × 3 cm plastic pots filled with “traysubstrat” soil and then returned to the Conviron^®^ for 15 days. Fifteen dpi seedlings were removed from the soil and thoroughly rinsed, taking care to keep the root system intact. The effect of the different *Fusarium* strains was subsequently assessed by measuring seedling (only aerial part) length (cm). Afterwards, the roots were cut off from the seedlings, freeze-dried by Heto Powder Dry LL3000 (Thermo Fisher Scientific), finely grounded with a Mixer Mill 400 (Retsch; 6 min, 25 Hz), and stored at −80°C for fungal biomass quantification as described in the ‘DNA extraction and *Fusarium* quantification by qPCR’ section The experiment was repeated twice and for each of the two experiments, nine independent seedlings, considered as replicates, per strain (or control) were carried out for a total of 54 seedlings. For qPCR analysis, three independent seedlings per strain or control were bulked into one replicate for DNA extraction, for a total of three replicates per strain or control.

#### *Fusarium* seedling stem base assay

Virulence assays on stem bases of common wheat seedlings were carried out according to a method previously reported [99] with several modifications [100]. Common wheat plants were grown in 6 × 8 × 8 cm plastic pots (5 seedlings per pot) in a growth chamber as described in the section on plant growth conditions. A 3 cm-long PVC collar (3 mm of internal diameter) was placed around the emerging coleoptiles. Seedlings were inoculated, at the fully extended second leaf stage (BBCH12 [98]). Inoculum was obtained by culturing the five *Fusarium* strains for one week at 22°C on 9-cm Petri dishes containing PDA (2 Petri dishes per strain). Subsequently, the fungal colony (together with the PDA) was homogenized in the Mixer Mill 400 (Retsch) with 12 mL of sterile water to obtain a homogenate of pipettable consistency. For control treatments, uninoculated PDA and sterile water were homogenised as described for the inoculum. Seedlings were inoculated by pipetting 700 µL of homogenate into the PVC collar. Seedlings were removed from the soil 15 dpi and rinsed thoroughly. Stem base infections were evaluated by measuring the length (cm) of the necrotic area and the presence of necrosis across leaf sheaths composing the stem base, using a 0–14 arbitrary scale (0 = clean; 1–2 = coleoptile; 3-4-5 = 1^st^ leaf; 6-7-8 = 2^nd^ leaf; 9-10-11 = 3^rd^ leaf; 12-13-14 = 4^th^ leaf) [99]. DI was calculated as the product between the average value (0–14) of necrosis across leaf sheaths and the average length (cm) of the necrotic area in the stem bases. Upon completion of disease evaluations, the first 5 cm of the stem base was cut, freeze-dried and finely ground as described in the *Fusarium* seedling root assay section, and stored at −80°C for DNA extractions and fungal biomass quantification. The experiment was repeated twice, each carried out with 15 independent seedlings (replicates) per strain, or control, for a total of 90 seedlings. For qPCR analysis, five independent seedlings per strain, or control, were bulked in one replicate for a total of three replicates.

#### Fusarium head assay

The head assay was performed as previously described [100] with several modifications. Common wheat plants were grown in the greenhouse in 9 × 9 × 13 cm pots (one seedling per pot) as described in the plant growth conditions section. Plants were watered regularly and fertilized at tillering (BBCH20 [98]), (using 3 g per pot of Nitrophoska® 13-10-20, EuroChem Agro SpA, Cesano Maderno, Italy). When the plants reached the beginning of the flowering stage (BBCH61) the main head was point inoculated with conidial suspensions of the different *Fusarium* strains, as previously described [101]. Inoculum was prepared using the same method described for the seedling root assay. Fifteen microliters of conidial suspension (1 × 10^6^ macroconidia mL^−1^) were injected between the palea and lemma of two adjacent spikelets near the center of the head. Inoculated spikelets were previously marked with a non-toxic permanent black marker to recognize them during the symptoms score. Immediately after the inoculation, the inoculated heads were covered for 48 h in clear plastic bags previously sprayed with water to maintain high humidity. After inoculation, plants were placed in a climatic chamber (F.lli Bertagnin, Bologna, Italy) at 22°C with a 16 h photoperiod and regularly watered. Mock-inoculated heads were used as controls.

FHB symptoms were evaluated at 7, 14, and 21 dpi, and assessed as the number of symptomatic spikelets out of the total number of spikelets per head, expressed as the percentage of the severity of symptoms (%). Wheat heads were hand-harvested at 28 dpi, freeze-dried by Heto Powder Dry LL3000 (Thermo Fisher Scientific) and finely ground by Mixer Mill 400 (Retsch). Three heads inoculated by each strain (or control) were bulked together and split into two subsamples. The first subsample was used to determine fungal biomass by qPCR, whereas the second one was used to quantify *Fusarium* secondary metabolites by LC-MS/MS, as mentioned before. The experiment was performed two times, with nine independent heads per strain or control, for a total of 54 heads each time. For qPCR analysis and LC-MS/MS analysis, three independent heads per strain or control were bulked in one replicate, resulting in three replicates per strain or control.

### DNA extraction and *Fusarium* quantification by qPCR

Total DNA was extracted from 20 mg of ground tissues collected from *Fusarium* virulence assay section using the CTAB method as previously described [102] with modifications [103]. DNA was quantified with a NanoDrop One (Thermo Fisher Scientific) and the concentration of each sample was adjusted to 25 ng µL^−1^. The qPCR assay was performed as described [103], using a CFX96 real-time PCR detection system (Bio-Rad, Hercules, California, USA). Species-specific primers (Table S6) were used for the quantification of *F. avenaceum* and of *F. graminearum* [104], whereas *translation elongation factor 1α* (*tef1α*) primers (Table S6) [102] were used for the quantification of wheat DNA (roots, stem bases, and heads). For standard curves, DNA was extracted from wild-type *F. avenaceum* and *F. graminearum* colonies (*Fa*WT and *Fg*WT, respectively) and healthy tissues (roots, stem base, and heads) of common wheat. Standard curves were generated as previously described [16, 104, 105] using a 10-fold dilution series of DNA ranging from 90 ng to 90 pg for wheat and 25 ng to 25 pg for *F. avenaceum* and *F. graminearum* in the seedling root assay. For the seedling stem base and head assays, the DNA ranging from 25 ng to 2.5 pg for *F. avenaceum* and *F. graminearum* and from 125 ng to 12.5 pg for wheat. The curves were generated by plotting the logarithmic values of known DNA quantities versus the corresponding cycle threshold (Ct) values and were performed for each assay. The *Fusarium* DNA in roots, stem bases, and heads were expressed as the ratio of the *Fusarium* DNA (pg) to the wheat DNA (ng).

### Statistical analysis

In vitro LC-MS/MS analysis was set up in a single experiment with three biological replicates. Virulence assays, for each of the two experiments, were set up in 9 (seedling length in root assay), 15 (DI in stem base assay), 9 (FHB symptoms in head assay), or 3 (qPCR from the root, stem bases, and heads; LC-MS/MS from heads) biological replicates. For the analysis of each of the virulence assays, two experiments were considered together and, except for in planta LC-MS/MS data, were log-transformed to normalize their distribution and homogenize the variances. As a consequence, the biological replicates increased to 18 (seedling length in root assay), 30 (DI in stem base assay), 18 (FHB symptoms in head assay), or 6 (qPCR from the root, stem bases and heads; LC-MS/MS from heads). Data are shown as their log-transformed average (two-experiment data except in planta LC-MS/MS data) or average (single-experiment data and in planta LC-MS/MS data) (± Standard Error). All data (log-transformed or not) were subject to one-way analysis of variance (ANOVA) by considering each “*Fusarium* strain” as the experimental factor and the different evaluated parameters as the variable. Finally, in the case of ANOVA significance (*p* < 0.05), to test pairwise contrasts, Tukey’s honestly significant difference multiple comparison tests were performed. All statistical analyses were performed using the Macro Excel^®^ “DSAASTAT” (version 1.0192; [106]).

### Electronic supplementary material

Below is the link to the electronic supplementary material.

Supplementary Material 1

Supplementary Material 2

## Data Availability

Data is provided within the manuscript or supplementary information files.
