# Supplementary material for: A comparison between the role of enniatins and deoxynivalenol in Fusarium virulence on different tissues of common wheat
Source: BMC Plant Biol. 2024 May 27;24:463. doi: 10.1186/s12870-024-04945-5 (PMC11129500; doi:10.1186/s12870-024-04945-5)
Supplement: Supplementary file 2 — Supplementary Material 2 [file 12870_2024_4945_MOESM2_ESM.docx]

**Supplementary material**

**A comparison between the role of enniatins and deoxynivalenol in *Fusarium* virulence on different tissues of common wheat**

Giovanni Beccari^1^, Francesco Tini^1,*^, Nora A. Foroud^2^, Luisa Ederli^1^, Donald M. Gardiner^3^, Aurelie H. Benfield^4^, Linda J. Harris^5^, Michael Sulyok^6^, Roberto Romani^1^, Ilaria Bellezza^7^, Lorenzo Covarelli^1^

*^1^Department of Agricultural, Food and Environmental Sciences, University of Perugia, Perugia, Italy;*

*^2^Lethbridge Research and Development Centre, Agriculture and Agri-Food Canada, Lethbridge, Canada;*

*^3^The University of Queensland, St. Lucia, Brisbane, Australia;*

*^4^School of Biomedical Sciences, Faculty of Health, Queensland University of Technology, Translational Research Institute, Brisbane, Australia;*

*^5^Ottawa Research and Development Centre, Agriculture and Agri-Food Canada, Ottawa, Canada;*

*^6^University of Natural Resources and Life Sciences, Vienna, Department of Agrobiotechnology (IFA-Tulln), Institute of Bioanalytics and Agro-Metabolomics, Tulln, Austria.*

*^7^Department of Medicine and Surgery, University of Perugia, Perugia, Italy;*

*Corresponding author: francesco.tini@unipg.it

**Table S1.** *In vitro* biosynthesis of other secondary metabolites (ng/g) produced by *Fusarium avenaceum* strains as detected by LC-MS/MS in autoclaved rice.

| **Secondary metabolites** |  | ***F. avenaceum* strain** | | |
| --- | --- | --- | --- | --- |
|  |  | ***Fa*WT** | ***Fa*Δ*esyn1*** | ***FaESYN1*_OX** |
| **Aurofusarin** | **ng/g** | 4.32 | 8.61 | 41.8 |
|  | **SE^a^** | 0.52 | 5.52 | 16.0 |
|  | **MCT^b^** | a | a | a |
| **Chrysogin** | **ng/g** | 802 | 230 | 813 |
|  | **SE** | 89.0 | 122 | 73 |
|  | **MCT** | a | b | a |
| **Moniliformin** | **ng/g** | 4530 | 260 | 5560 |
|  | **SE** | 520 | 202 | 617 |
|  | **MCT** | a | b | a |

^a^SE = ± Standard Error; ^b^MCT = Multiple Comparison Test

**Table S2.** *In vitro* biosynthesis of other secondary metabolites (ng/g) produced by *Fusarium graminearum* strains as detected by LC-MS/MS in autoclaved rice.

| **Secondary metabolites** |  | ***F. graminearum* strains** | |
| --- | --- | --- | --- |
|  |  | ***Fg*WT** | ***Fg*Δ*tri5*** |
| **15-Hydroxyculmorin** | **ng/g** | 47200 | 5390 |
|  | **SE^a^** | 11900 | 404 |
|  | **MCT^b^** | a | b |
| **5-Hydroxyculmorin** | **ng/g** | 36100 | 7870 |
|  | **SE** | 4970 | 637 |
|  | **MCT** | a | b |
| **15-Hydroxyculmoron** | **ng/g** | 3330 | 394 |
|  | **SE** | 1170 | 36.2 |
|  | **MCT** | a | a |
| **Culmorin** | **ng/g** | 11400 | 3950 |
|  | **SE** | 2270 | 743 |
|  | **MCT** | a | a |
| **Aurofusarin** | **ng/g** | 285 | 205 |
|  | **SE** | 105 | 119 |
|  | **MCT** | a | a |
| **Butenolide** | **ng/g** | 1020 | 337 |
|  | **SE** | 550 | 117 |
|  | **MCT** | a | a |
| **DON-3-glucoside** | **ng/g** | 17700 | <LOD^c^ |
|  | **SE** | 2840 | <LOD |
|  | **MCT** | a | b |

^a^SE = ± Standard Error; ^b^MCT = Multiple Comparison Test; ^c^LOD = Limit of Detection

**Table S3.** Results of FHB symptom observation. See Excel file.

**Table S4.** Secondary metabolites (ng/g) other than ENNs detected in common wheat heads at 28 dpi following head inoculation with *F. avenaceum* strains.

| **Secondary metabolites** |  | ***F. avenaceum* strain** | | |
| --- | --- | --- | --- | --- |
|  |  | ***Fa*WT** | ***Fa*Δ*esyn1*** | ***FaESYN1*_OX** |
| **Aurofusarin** | **ng/g^a^** | 436 | 563 | 84.0 |
|  | **SE^b^** | 355 | 343 | 27.7 |
|  | **MCT^c^** | a | a | a |
| **Chrysogin** | **ng/g** | 847 | 442 | 631 |
|  | **SE** | 100 | 145 | 122 |
|  | **MCT** | a | a | a |
| **Moniliformin** | **ng/g** | 4830 | 1180 | 4080 |
|  | **SE** | 368 | 556 | 861 |
|  | **MCT** | a | a | a |

^a^average of two independent experiments (each composed of three replicates, each composed of three heads bulked together); ^b^SE = ± Standard Error; ^c^MCT = Multiple Comparison Test

**Table S5**. Secondary metabolites (ng/g) other than trichothecene detected in common wheat heads at 28 dpi following head inoculation with *F. graminearum* strains.

| **Secondary**  **metabolites** |  | ***F. graminearum* strains** | |
| --- | --- | --- | --- |
|  |  | ***Fg*WT** | ***Fg*Δ*tri5*** |
| **15-Hydroxyculmorin** | **ng/g^a^** | 47200 | 11900 |
|  | **SE^b^** | 5390 | 404 |
|  | **MCT^c^** | a | b |
| **5-Hydroxyculmorin** | **ng/g** | 36100 | 7870 |
|  | **SE** | 4980 | 637 |
|  | **MCT** | a | b |
| **15-Hydroxyculmuron** | **ng/g** | 3330 | 394 |
|  | **SE** | 394 | 36.2 |
|  | **MCT** | a | b |
| **Culmorin** | **ng/g** | 11400 | 2270 |
|  | **SE** | 3950 | 743 |
|  | **MCT** | a | b |
| **Aurofusarin** | **ng/g** | 285 | 105 |
|  | **SE** | 205 | 119 |
|  | **MCT** | a | a |
| **Butenolide** | **ng/g** | 1020 | 337 |
|  | **SE** | 549 | 117 |
|  | **MCT** | a | b |
| **Chrysogin** | **ng/g** | 996 | 614 |
|  | **SE** | 167 | 16.6 |
|  | **MCT** | a | a |
| **Deoxynivalenol-**  **3-glucoside** | **ng/g** | 17700 | <LOD^d^ |
|  | **SE** | 2840 | <LOD |
|  | **MCT** | a | b |

^a^average of two independent experiments (each composed of three replicates, each composed of three heads bulked together); ^b^SE = ± Standard Error; ^c^MCT = Multiple Comparison Test; ^d^LOD = Limit of Detection

**Table S6.** Barcoding sequence and primers used in the realization of *FgΔtri5* strain.

| **Name** | **Target** | **Sequences** | **References** |
| --- | --- | --- | --- |
| **Barcode** | Synthetic sequence | GATGTCCACGAGGTCTCTAGTAGCGCGTT  CAATCTAGCCGTACGCTGCAGGTCGAC | - |
| **NATr primer** | Nourseothricin acetyl transferase gene | 5'-CGTGTCGTCAAGAGTGGTCA-3' | 92 |
| **TRI5promF** | *TRI5* upstream region | 5'-TTCGCATTGACTTTGGATCA-3' | - |
| **Tri5cdsR** | *TRI5* coding sequence | 5'-GGCAGCCTTGTTGTAAGCAT-3' | - |
| **Fave574 fwd** | *F. avenaceum* | 5’-TATGTTGTCACTGTCTCACACCACC-3’ | 101 |
| **Fave627 rev** |  | 5’-AGAGGGATGTTAGCATGATGAAG |  |
| **Fg16N fwd** | *F. graminearum* | 5’-ACAGATGACAAGATTCAGGCACA-3’ | 101 |
| **Fg16N rev** |  | 5’-TTCTTTGACATCTGTTCAACCCA-3’ |  |
| **Hor1 fwd** | Wheat (*tef1α*) | 5’-TCTCTGGGTTTGAGGGTGAC-3’ | 101 |
| **Hor2 rev** |  | 5’-GGCCCTTGTACCAGTCAAGGT-3’ |  |

**Table S7.** LODs and LOQs in ng/g for deoxynivalenol and enniatins, re-determined for processed wheat matrices according to EURACHEM.

| **Secondary metabolites** | **LOD (ng/g)** | **LOQ (ng/g)** |
| --- | --- | --- |
| **Deoxynivalenol** | 1.20 | 3.90 |
| **Enniatin A** | 0.01 | 0.04 |
| **Enniatin A1** | 0.03 | 0.10 |
| **Enniatin B** | 0.02 | 0.05 |
| **Enniatin B1** | 0.03 | 0.10 |
| **Enniatin B2** | 0.02 | 0.07 |
| **Enniatin B3** | 0.0004 | 0.0012 |


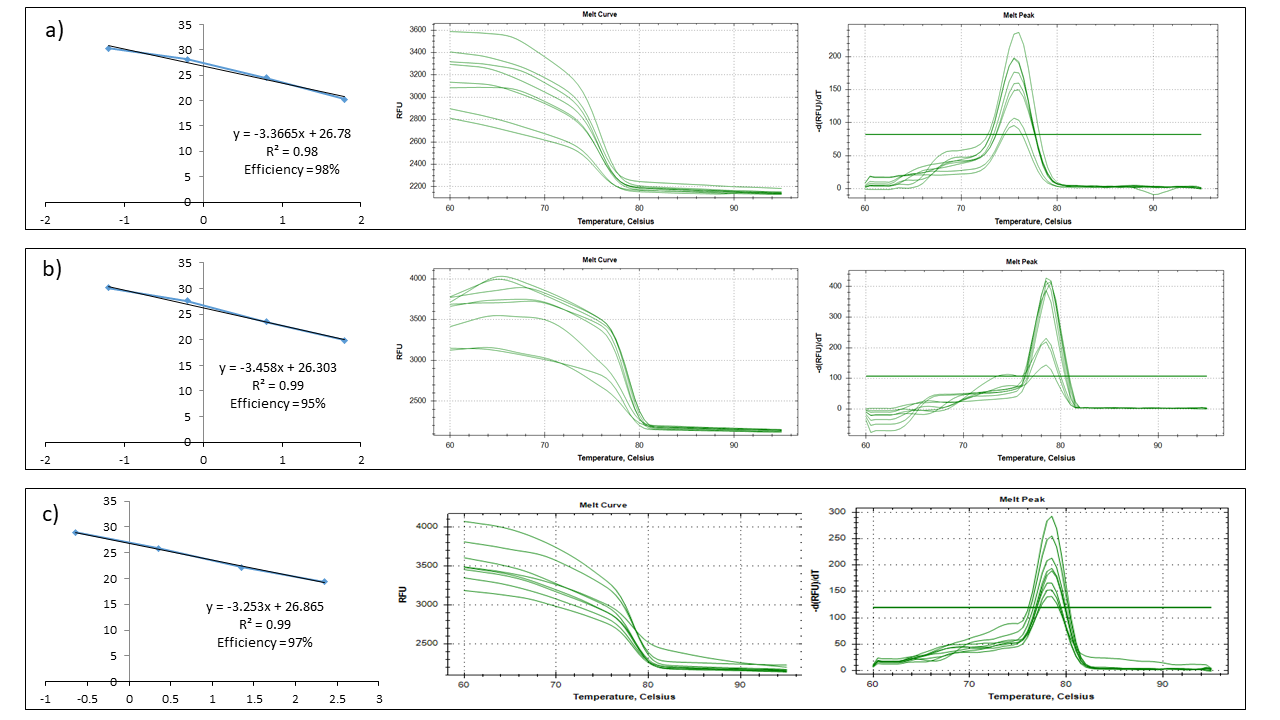


**Figure S1.** Standard curves with linear equations, R^2^ values and efficiencies, as well as melting curves and melt peak of *F. avenaceum* (a), *F. graminearum* (b), and wheat (c) obtained by qPCR and used in the *Fusarium* seedling root rot assay.


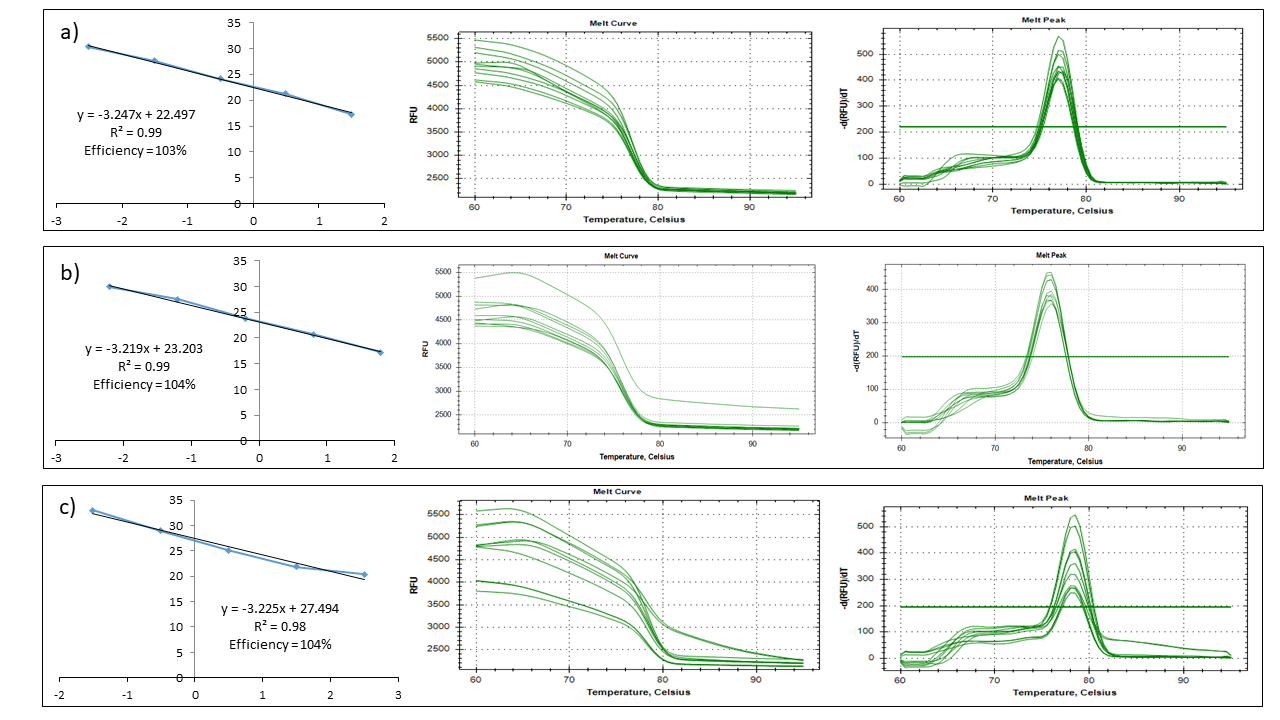


**Figure S2.** Standard curves with linear equations, R^2^ values and efficiencies, as well as melting curves and melt peak of *F. avenaceum* (a), *F. graminearum* (b), and wheat (c) obtained by qPCR and used in the *Fusarium* seedling stem base assays.


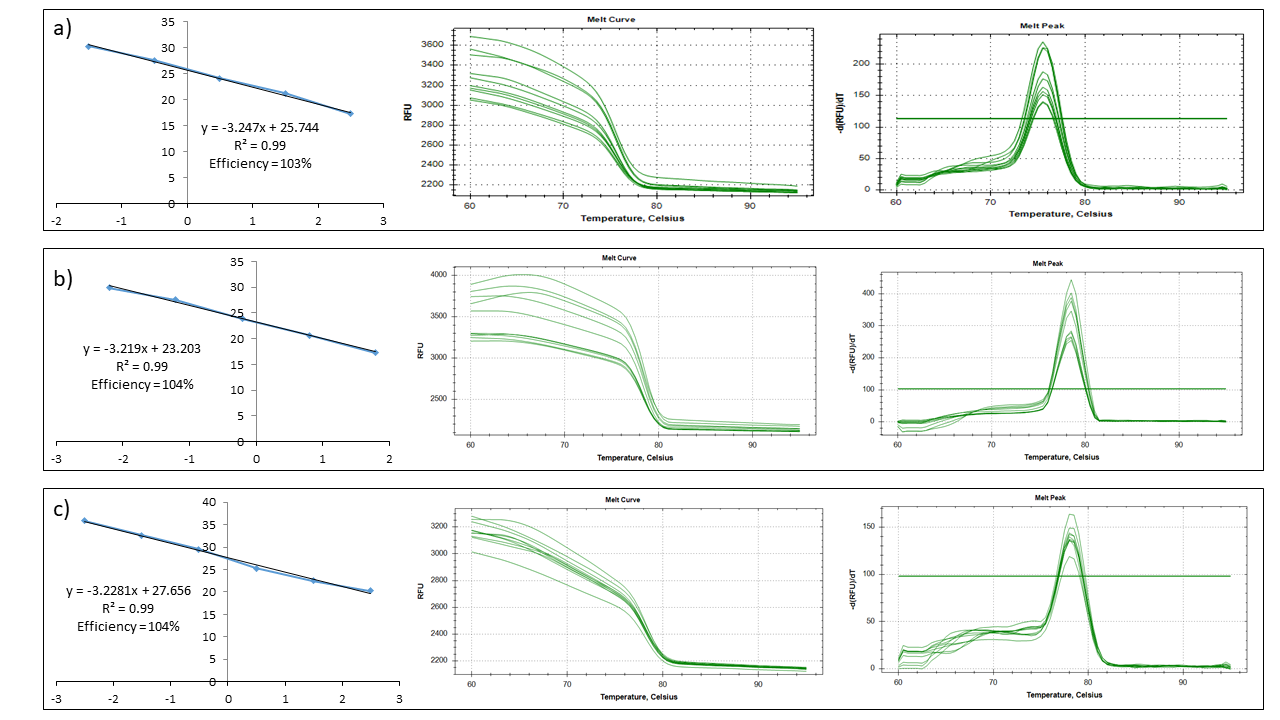


**Figure S3.** Standard curves with linear equations, R^2^ values and efficiencies, as well as melting curves and melt peak of *F. avenaceum* (a), *F. graminearum* (b), and wheat (c) obtained by qPCR and used in the Fusarium head blight assays.
